# Supplementary material for: 5-Hydroxytryptamine Enhances the Pacemaker Activity of Interstitial Cells of Cajal in Mouse Colon
Source: Int J Mol Sci. 2024 Apr 3;25(7):3997. doi: 10.3390/ijms25073997 (PMC11012597; doi:10.3390/ijms25073997)
Supplement: Supplementary file 1 [file ijms-25-03997-s001.zip › ijms-2905962-supplementary.pdf]

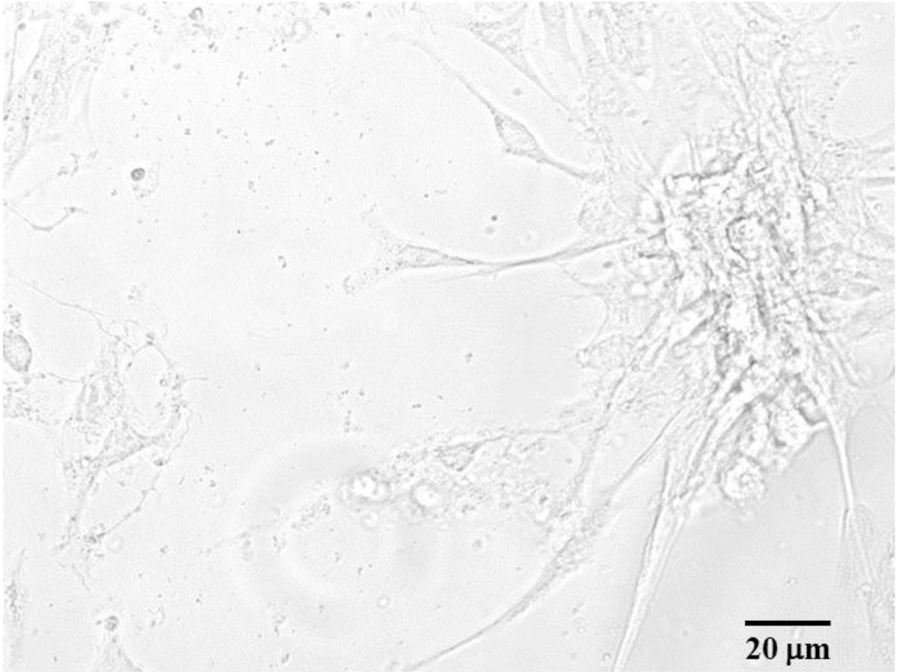

Supplementary Figure S1. Cultured ICCs from mouse colon. Light microscope image of a colonic ICC network.
